# How to Target Small-Molecule Fluorescent Imaging Probes to the Plasma Membrane—The Influence and QSAR Modelling of Amphiphilicity, Lipophilicity, and Flip-Flop

**DOI:** 10.3390/molecules28227589

**Published:** 2023-11-14

**Authors:** Richard W. Horobin, Juan C. Stockert

**Affiliations:** 1 Chemical Biology and Precision Synthesis, School of Chemistry, University of Glasgow, Glasgow G12 8QQ, UK; 2 Instituto de Ciencias Ambientales y Salud, Fundación PROSAMA, Paysandú 752, Buenos Aires CP1405, Argentina; jcstockert@prosama.com.ar; 3 Centro Integrativo de Biología y Química Aplicada (CIBQA), Universidad Bernardo O’Higgins, General Gana 1702, Santiago 8370854, Chile

**Keywords:** design, fluorescent probe, plasma membrane, QSAR

## Abstract

Many new fluorescent probes targeting the plasma membrane (PM) of living cells are currently being described. Such probes are carefully designed to report on relevant membrane features, but oddly, the structural features required for effective and selective targeting of PM often receive less attention, constituting a lacuna in the molecular design process. We aim to rectify this by clarifying how the amphiphilicity and lipophilicity of a probe, together with the tendency to flip-flop across the membrane, contribute to selective PM accumulation. A simplistic decision-rule QSAR model has been devised that predicts the accumulation/non-accumulation of small-molecule fluorescent probes in the PM. The model was based on probe log P plus various derived measures, allowing the roles of amphiphilicity, lipophilicity, and flip-flop to be taken into account. The validity and wide applicability of the model were demonstrated by evaluating its ability to predict amphiphilicity or PM accumulation patterns in surfactants, drugs, saponins, and PM probes. It is hoped that the model will aid in the more efficient design of effective PM probes.

## 1. Introduction

Fluorescent probes targeting the plasma membrane (PM) are currently a hot topic and have been addressed in several recent reviews, e.g., [1,2,3]. Such probes are used for identifying the PM and also to report on certain of its properties, such as membrane potential and viscosity. Indeed, PM probes can also report on the properties of membrane adjacent regions inside or outside the cell (e.g., Ca^++^, pH). Finally, probes can report on biological phenomena involving this membrane (e.g., apoptosis, endocytosis). To achieve such varied applications, PM probes exhibit a wide diversity of molecular character. The current article, however, is focused on the restricted category of small-molecule fluorescent probes targeting the lipid domain of the PM. Consequently, reactive probes, whether attaching to lipids or proteins, are not considered here.

Even when considering only this restricted group of probes, many compounds have been devised, and much effort has gone into their design. Curiously, the chemical factors regulating localisation in the PM of live cells have received relatively little attention. When the topic is discussed, there is often a general emphasis on the presumed need for lipophilicity. As will be seen, this is significantly inadequate. Consequently, we here seek to clarify the interrelated roles of amphiphilicity, lipophilicity, and flip-flop in such PM-targeted reagents. In particular, we show how these widely relevant properties can be modelled by simplistic decision-rule quantitative structure–activity relations (QSAR) models.

Lipophilicity is the preference of a molecule or molecular domain to localise in a lipophilic environment. This is a conceptually imprecise term describing a biologically significant property. The overall lipophilicity of a probe is usually parameterized by the partition coefficient of the molecule between water and some non-polar organic solvent, typically octanol, e.g., [4,5]. A lipophilic molecule may be composed of a single hydrophobic domain, with the molecule’s log P > 0. As has long been known [6], a lipophilic probe will partition into membranes. Moreover, if a molecule is applied to a cell from an external solution, then the PM is the first such target the probe will meet. Of course, much sophisticated physicochemical work and conceptual comment have been carried out in this arena—e.g., [7,8]—but in the present account, a simplistic approach is adopted.

Amphiphilicity is a property of molecules possessing both hydrophilic and lipophilic domains. These regions have sometimes been termed the “headgroup” (HG) and the “tail,” respectively. Amphiphilicity is a distinct property influencing the interactions of small-molecule probes with biomembranes, quite independently of overall lipophilicity. Indeed, the overall character of an amphiphile may be either lipophilic or hydrophilic. For instance, if a hydrophilic molecule, which does not partition into a membrane, is amphiphilic, then the lipophilic domain may insert into the membrane, leaving the hydrophilic HG in an external aqueous region. Amphiphilicity has also been much studied by physical chemists. There is, for instance, an extensive account of the prediction of surface activity in chapter 15 of the monograph of Rosen and Kunjappu [9]. Again, such sophisticated approaches are not discussed in the present account.

Next, consider the loss of a probe previously taken into a membrane from an external medium. This loss may occur either by the probe returning to the external medium or by its permeation into the cell interior. Regarding partitioning, unless the probe is trapped within the lipophilic domain of the membrane, it will indeed bleed out in this way. However, with probes inserted into the membrane due to amphiphilicity, the situation is more complicated. Thus, if the HG is not too hydrophilic or too large, the molecule might then flip-flop across the membrane to the inner leaflet, from where it might enter the cell interior [10].

The use of QSAR models to describe the interactions of small-molecule fluorescent probes with various cell membrane systems has been addressed previously by the present authors [11]. This earlier account, however, did not focus on the PM and so lacked both detail and context regarding this organelle. Moreover, the core QSAR models for membrane uptake were based on a relatively small number of probes. Consequently, in the present account, we aim to provide more detail and an integrated PM-specific QSAR model. This is then critically evaluated with the aid of a larger sample of compounds.

In addition to the restriction of the present account to small-molecule probes, certain other limitations should be pointed out. The model does not address PM derivatives such as exosomes and endosomes, nor does it allow for the fact that PM is sometimes relatively fluid (e.g., in spermatozoa [12]) and can vary in permeability (e.g., during the cell cycle [13]). Finally, it must be acknowledged that reliable estimates of the necessary structure parameters (AI, HGH, and log P, see below) cannot be made for certain types of chemical structures.

## 2. Results

Estimated structure parameters for each of the test-case sets of compounds are provided in Table 1, Table 2, Table 3, Table 4 and Table 5. The chemical diversity of these compounds is illustrated by the structural formulae of a sample of these listed compounds given in Figure S1.

Table 1 contains information on 17 commercial surfactants. The HGs of these compounds are listed and show considerable diversity; see structures A and B in Figure S1. The lipophilic domains have less variation, mostly being saturated linear hydrocarbons of varying chain lengths.

Table 2 contains structure parameter information on 16 drugs considered to display surface-active properties. These agents are used to treat a wide range of conditions, as indicated by the information on “types of drugs.” The drugs are mostly amine salts with a few weak acids. The overall chemical character of the molecules shows considerable diversity; see structures C and D in Figure S1.

Table 3 contains structure parameters for 13 saponins. These strongly amphiphilic natural products provide a dramatic structural contrast with the typically synthetic compounds in Table 1 and Table 2. The saponin HGs comprise complex sugar chains, or amino, carboxylic acid, or hydroxyl moieties. The lipophilic domains are steroidal or triterpenoid, sometimes with aromatic or alicyclic substituents. Exemplary structures are given in panels E and F of Figure S1.

Table 4 contains structure parameters for 46 PM probes. The various membrane targets are also tabulated. The HGs are varied and may be anionic (e.g., carboxylate, phosphate, or sulfonate), cationic (e.g., amine salts, quaternary salts), or neutral (e.g., amido, polyether, zwitterionic). The lipophilic domains are also diverse, with a variety of aromatic and heteroaromatic skeletons, sometimes carrying hydrocarbon chains as substituents. Exemplary structures are given in panels G and H of Figure S1.

Table 5 contains structure parameters for a set of FM and styryl dyes investigated by [17] for use in assessing endo- and exocytosis. The activity dependence and certain staining characteristics described by these authors are also tabulated.

**Table 5:** Various structural parameters of plasma membrane probes of the FM or styryl type. Examples from [18]. Tabulation follows the sequence of Table 1 in that paper; all listed structures that meet the selection criteria are given below. AI: amphiphilicity index. Log P: log of the octanol–water partition coefficient. Activity dependence increases from − to ++++.

Dye Name	Structure Parameters		Activity Dependence
	AI	Log P	
FM 14-68	2.2	−2.5	+
FM 2-10	2.7	−2.0	++
FM 1-43	4.2	−0.5	++++
FM 4-84	4,2	−3.3	++++
FM 1-84	5.0	0.3	++++
FM 14-27	6.6	1.9	−
FM 14-29	8.2	3.5	−
FM 3-25	15.9	11.2	−
RH 414	3.4	−1.3	+++
FM 6-55	4.2	−0.5	+++
FM 10-75	3.6	−1.1	++
FM 4-64	4.1	−0.6	++++
FM 1-81	5.7	1.0	−
FM 9-49	2.5	−0.5	−
FM 4-95	5.3	0.6	−
FM 5-27	7.0	−2.4	−
FM 4-59	1.3	−3.4	−

## 3. Discussion

### 3.1. Clarifications Concerning Amphiphilicity and Lipophilicity

**Figure 1:**
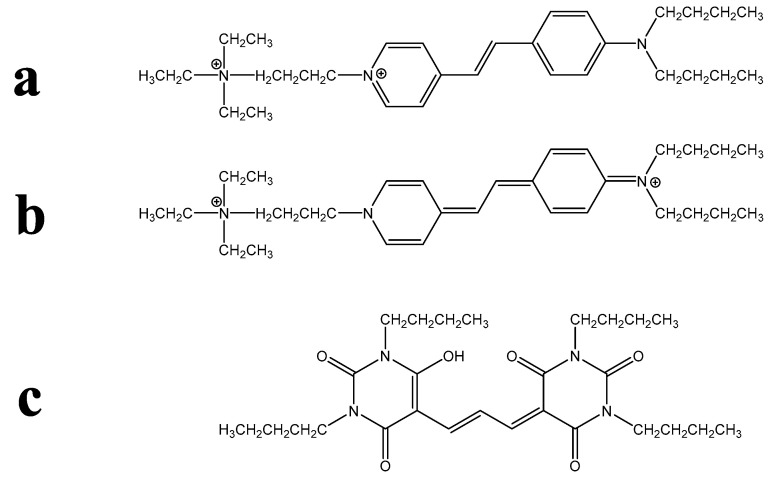
Examples of chemical formulae that may be problematic for non-chemists. (**a**) The most commonly illustrated resonance form of the PM probe is FM 1-43, in which amphiphilicity is clearly indicated. (**b**) Another resonance form of FM 1-43 gives a different impression of amphiphilic possibility. (**c**) The usual, two-dimensional representation of the PM probe DiBAC4(3), in which the lipophilic butyl substituents are shown in the same plane as the hydrophilic molecular core.

Despite amphiphilicity not being a synonym for lipophilicity, these two terms are nevertheless sometimes confused in the biological literature. Another difficulty for non-chemists is the interpretation of structural chemical formulae. For instance, the structures of the widely used PM-binding FM dyes and their analogues are commonly drawn in a way that exaggerates their amphiphilic character, as is shown for FM 1-43 in Figure 1a. Biologists may not appreciate that such a structure represents a single resonance form, whereas the charge may actually be delocalised across the molecule; see Figure 1b for the depiction of a second resonance form of FM 1-43, which gives a different visual impression of the amphiphilic character of the dye. Another potential confusion arises when two-dimensional structural formulae are used to represent non-planar molecules. For instance, consider the fluorescent membrane probe DiBAC4(3), shown in Figure 1c. A non-chemist may not appreciate that the four butyl substituents can bend out of the plane of the hydrophilic aromatic core—and out of the plane of the diagram on the page—to generate a strongly lipophilic domain.

A final structural complication is problematic for biologists and chemists alike. This arises with dyes likely to be present as more than a single ionic species under physiological conditions or, indeed, when resonance structures differ markedly in an analogous way. This can make the estimation of log P imprecise. Such phenomena can arise with zwitterionic solvatochromic dyes as well as with oxazones, rhodols, thiazones, etc.; for potential examples, see [18,19,20].

### 3.2. Core QSAR Models: Describing Membrane Uptake of Probes

As noted above, the lipophilicity/hydrophilicity of a probe has typically been parameterized using a partition coefficient, typically between octanol and water. This is usually reported as the log P_oct_, log Pow, or merely the log P value of the probe. Such values are sometimes measured [5] but very often estimated in various ways, examples of the latter being the fragment procedures developed by Rekker [21] and Hansch and Leo [4,22]. Prior simplistic decision-rule QSAR models for staining of biomembranes by probes [11] utilised the Hansch and Leo procedure and regarded a probe whose log P > 0 as lipophilic and potentially membrane permeant. When log P > 5, a probe is expected to first accumulate in but not remain trapped within the membrane. However, when log P > 8, a probe will be effectively trapped in the first membrane encountered. Such models are to be regarded as simplistic since they ignore some significant physicochemical phenomena. For instance, in the case of ionic probes, the nature of the counter ion can dramatically influence permeability [23].

Probe amphiphilicity has also previously been assessed by a simplistic QSAR modelling approach [11]. This approach considered that the insertion or otherwise of the lipophilic domain of an amphiphilic probe into a biomembrane would correlate with the nominal log P of the probe’s lipophilic domain (the amphiphilicity index; AI). If AI < 3.5 log P units, no membrane accumulation will occur, while AI values exceeding 5 or 8 log P units correspond, respectively, to the occurrence of significant accumulation in, or effective trapping within, a biomembrane. Analogously, the HG requires minimal hydrophilicity (HGH < −1 log P units) for the HG to remain at the membrane surface or to protrude into the aqueous phase while the lipophilic domain is inserted in the hydrophobic membrane core. The HGH is again a nominal log P value, but this time for the hydrophilic probe domain.

However, an integration of these models and of the use of the AI, HGH, and log P structure parameters to specifically address the features of PM probes is not available. Consequently, this is the next step taken here.

### 3.3. An Integrated QSAR Model of PM Probes

**Figure 2:**
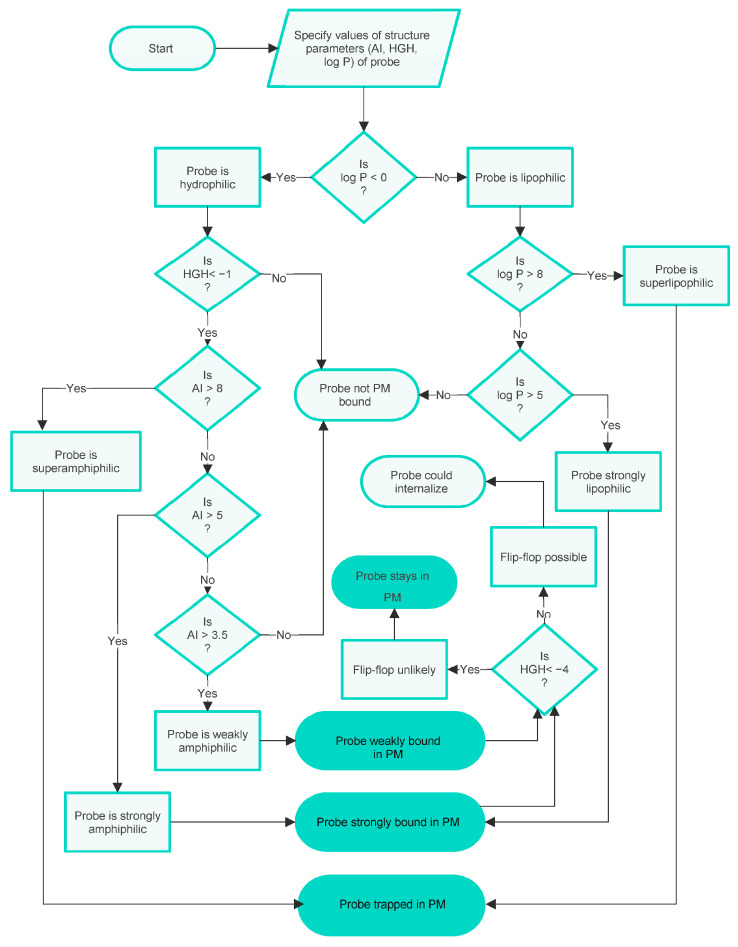
A flowchart specifying the integrated decision-rule QSAR model of PM staining by small-molecule fluorescent probes shows the influences of amphiphilicity, lipophilicity/hydrophilicity, and flip-flop. This version of the model disregards possible PM internalisation.

Although the parameter cut-off values noted above are few in number, their combinations predict several distinct membrane localisations and several strengths of membrane binding for interactions of probes with the PM of a live cell. The logic of this integration is assembled in the form of a flowchart in Figure 2.

It can be seen from this chart that the possible outcomes predicted for probes with various combinations of physicochemical characteristics are as follows:

1. To be effectively trapped in the PM.
2. To be strongly but not irreversibly bound to the PM, so able to internalise via flip-flop.
3. To be weakly bound to the PM, so able to internalise via flip-flop.
4. Not bound to the PM.

To summarise, the integrated model visualised in the flowchart in Figure 2 predicts three types of PM binding. It also predicts when entry into the cell interior can occur and gives some indication of the strength of binding, or lack of binding, to the PM. These conclusions are based on the cut-off values of the parameters AI, HGH, and log P describing the probes, which reflect the physicochemical character of the probes.

At this point, it should be reiterated that the model is simplistic and that there are additional factors that can, on occasion, complicate such predictions. For instance, consider Zn-tetramethyl-2,3-pyridinium-porphyrazine, whose major species is strongly hydrophilic and so membrane impermeant. Yet this dye does enter cells, as evidenced by the staining of mitochondria [24]. Probably, the dye has been partly reduced or converted to amphiphilic pseudobases, which can enter live cells by flip-flop.

**Figure 3:**
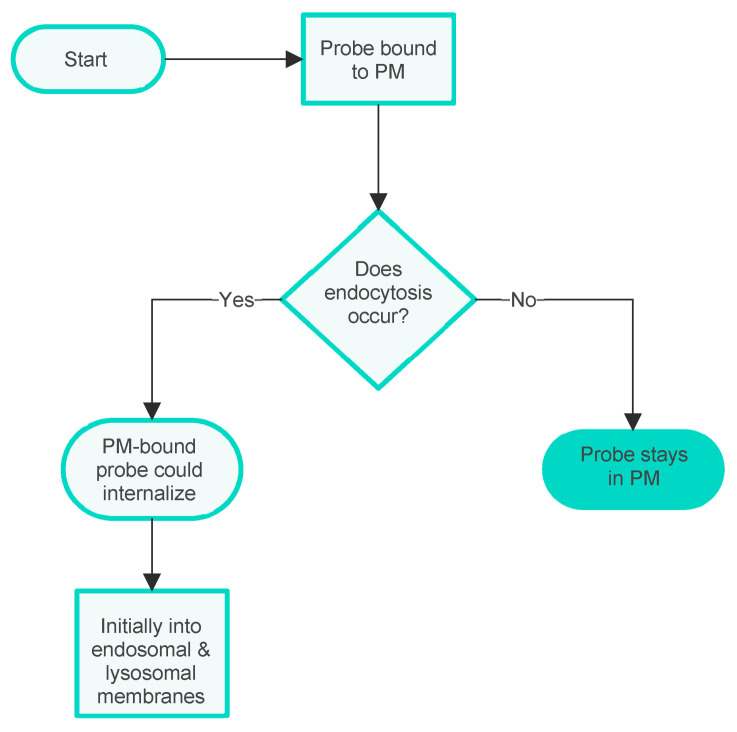
Flowchart showing the influence of membrane internalisation on the internalisation of PM probes into the cell interior.

Another biological complication is also sometimes significant for PM probe accumulation. Namely, the possibility that the PM is taken into the cell during an endocytic process, carrying with it any bound probe. There are several distinct processes of this kind [25], but this complexity will not be discussed here as it does not significantly affect the arguments presented. At this point, it is sufficient to note that in an experimental situation, the possibility of membrane internalisation can be manipulated, e.g., by lowering the temperature of the experimental system to prevent endocytosis [26] or by the administration of inhibitors of the various processes [27]. Possible additional outcomes of PM internalisation are indicated in the additional flowchart given in Figure 3.

**Figure 4:**
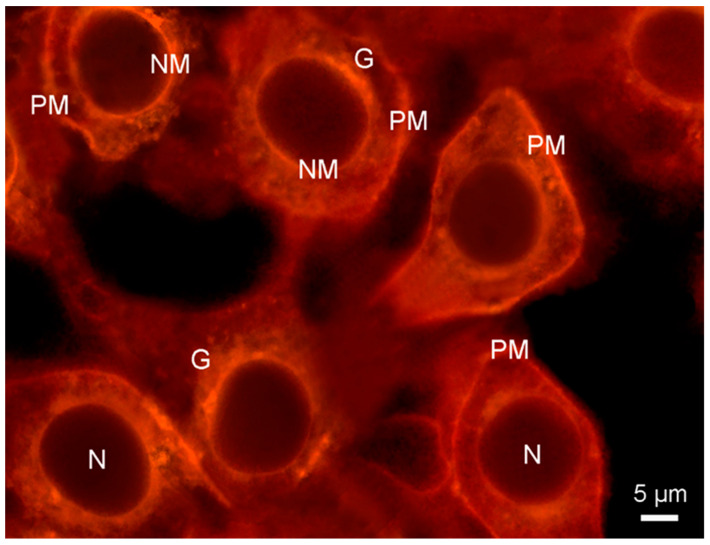
Fluorescence micrograph showing plasma membrane and other organelle membranes of cultured HeLa cells visualised with merocyanine 540 (20 μM in DMEM medium for 5 min, double blue-green exciting light: 460/490 + 510/550 nm), followed by a washing step with DMEM for 10 min. Substantial probe internalisation occurred after 15 min, with only the membrane-free nuclei (N) remaining unstained. G: Golgi. NM: nuclear membrane. PM: plasma membrane.

An example of these processes is provided by merocyanine 540. This dye has been used as a PM stain and, indeed, was an early membrane potential probe [28]. However, due to its giving rise to photodamage and its ease of internalisation, the latter being illustrated in Figure 4, merocyanine 540 has been replaced by other dyes for such applications.

**Figure 5:**
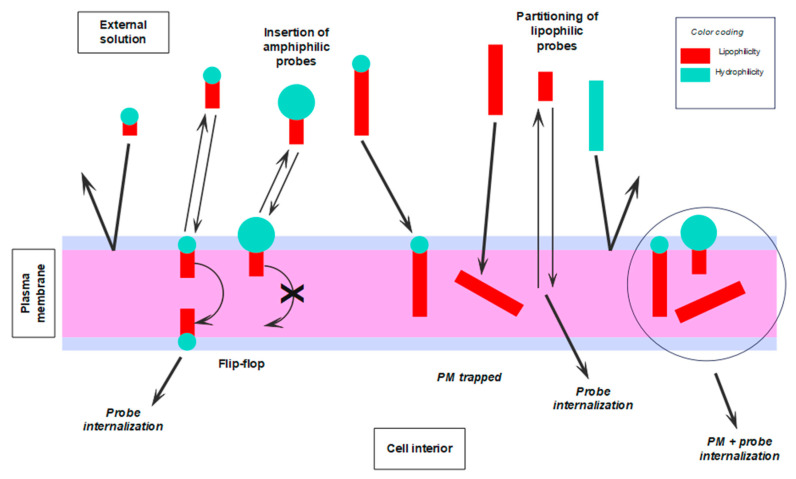
Visual mnemonics illustrate some of the factors, physicochemical and biological, influencing the interaction of fluorescent probes with the PM of living cells and the subsequent fate of such probes. Graphical coding: the larger the blue circles and red rectangles, the more hydrophilic the HG and the more lipophilic the tail, respectively.

Finally, in Figure 5, we give a visual mnemonic illustrating common factors influencing the binding of fluorescent probes to the PM of living cells, as well as the subsequent fates of the different types of dye.

### 3.4. Assessing the QSAR PM Staining Model and Its Cut-Off Values

The fact that the integrated PM staining model can be described using a flowchart indicates that it possesses internal coherence, which is reassuring. The underlying core QSAR models, however, originated from a consideration of rather small sets of probes. Hence, the broader applicability of the model needed to be assessed. First, by obtaining non-cherry-picked sets of known surface active compounds of various types, estimating their appropriate structure parameters, and then checking to see if they were compliant with the cut-off values of the models. Second, by obtaining a set of known PM localising probes and checking those out in a similar manner. Relevant structure parameters (i.e., AI, HGH, log P, and Z) were estimated for these sets of probes. As indicated by Figure 2, the fine-grained prediction made by the model meant that this tactic provided several tests of the model’s validity.

**Figure 6:**
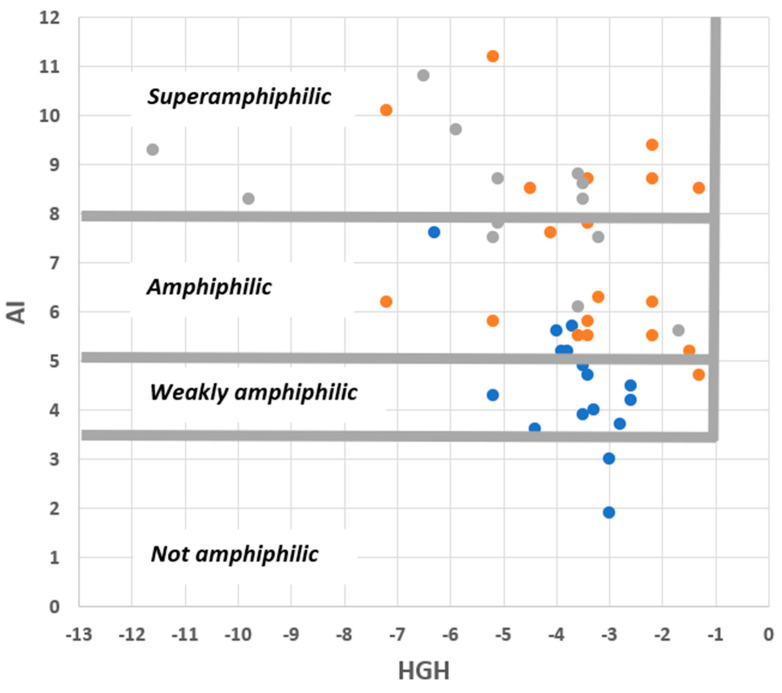
Distribution of commercial surfactants (orange circles), pharmaceutical agents (blue circles), and saponins (grey circles) on an AI–HGH chart. The features specified are those predicted by the integrated QSAR model to result in amphiphilicity.

With this in mind, first inspect Figure 6, in which sets of commercial surfactants, pharmaceutical agents, and saponins are plotted onto an AI-HGH chart. It is apparent that the cut-off values of the integrated model are appropriate in a large majority (46/48) of cases. The chemical character of the compounds plotted is very variable (see Table 1, Table 2 and Table 3), so this is a reassuring finding. Moreover, it is noteworthy that commercial surfactants and saponins, whose roles require amphiphilicity, all fall into the “amphiphilic” or “superamphiphilic” regions of the chart. This can be contrasted with pharmaceutical agents, whose amphiphilic character is typically coincidental with their function and whose compounds mostly fall into the “weakly amphiphilic” region.

**Figure 7:**
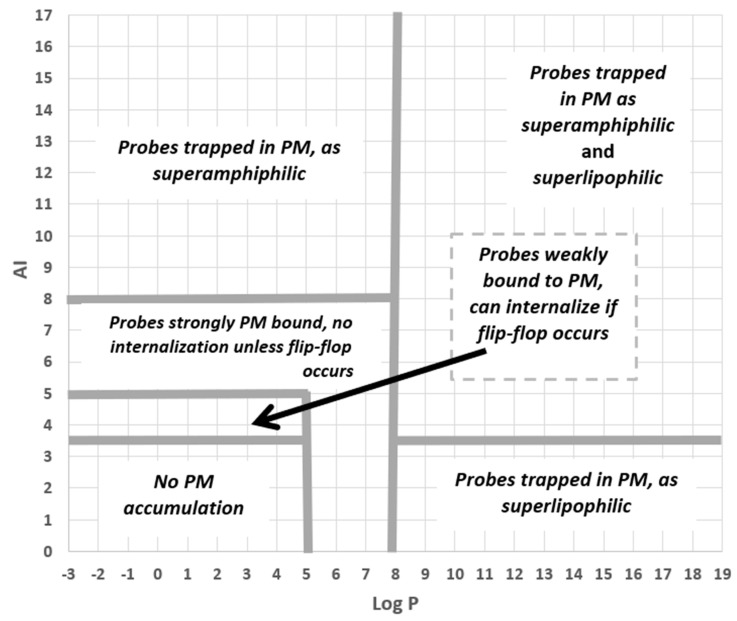
The different probe-PM binding mechanisms and binding strengths, as predicted by the QSAR model, are shown as separate regions of an AI–Log P chart.

**Figure 8:**
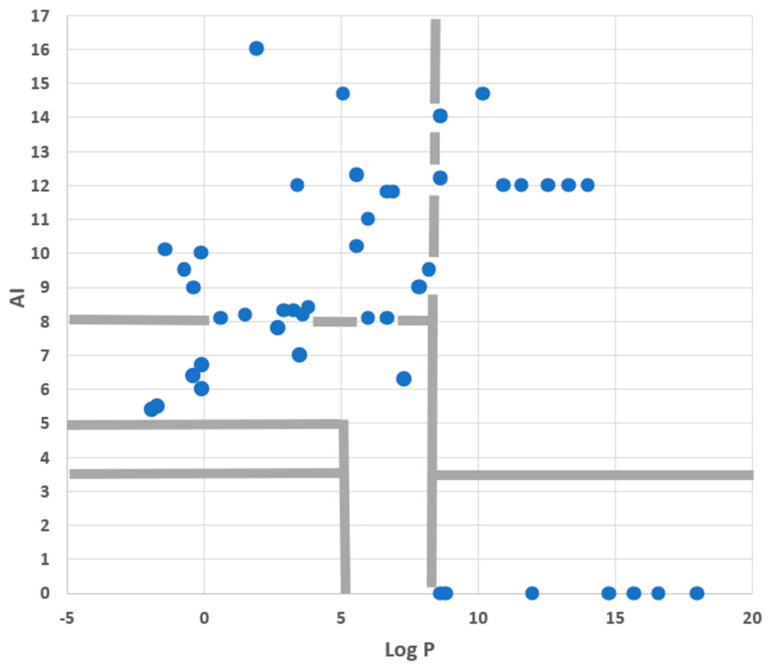
A non-cherry-picked set of small-molecule fluorescent probes targeting PM was plotted onto an AI–Log P chart. The separate regions of the diagram are identified in Figure 7. More complete information on these probes is provided in Table 4.

Finally, we looked at the set of fluorescent PM probes listed in Table 4 with two issues in mind. First, consider the roles of amphiphilicity, lipophilicity, and flip-flop directly in the case of fluorescent probes. Second, to check on which mechanisms were involved with the PM accumulation of these compounds. To accomplish this, we used an AI-Log P chart since, as seen in the annotated version of this diagram given in Figure 7, this provides a direct visual identification of six mechanistic categories involving amphiphilicity and lipophilicity while also linking to the role of flip-flop. As Figure 8 shows, PM probes fall into most mechanistic categories, and, not surprisingly, most probes are either superamphiphilic or superlipophilic. The exploitation of superamphiphilicity is particularly striking. This perhaps reflects the fact that superlipophilic compounds are typical of low aqueous solubility and require technical ingenuity to facilitate their solubilisation in cell-compatible solutions, e.g., [29].

Given that the dyes plotted in Figure 8 were all taken from a review of recent PM probes, it is obviously no surprise, albeit reassuring for the present analysis, that none of them fall into the “No PM accumulation” region of the diagram. However, the lack of probes in the “Probes weakly bound to PM” region does call for comment. This absence is almost certainly due to sampling bias, arising from the fact that the review article [1] providing the dyes plotted in Figure 8 emphasised recent research rather than routine but older probes. However, for certain investigations, there has been, and still is, a requirement for weakly bound PM probes. This tactic used, for instance, to assist in the study of exo- and endocytosis occurring during the secretion of neurotransmitters [30] or during the formation of endosomes in plants [31]. In such work, the dye must be removed from the PM after exo- or endocytosis has taken place.

To assess whether the physicochemical features of probes used in such studies are, in fact, congruent with the QSAR model described here, we used information summarised in, or derived from, a review describing the development of the so-called FM dyes [17]. This showed—see Table 5—that the six best probes (of 17 investigated) all fell within or on the border of the “probes weakly bound to PM” region of an AI-Log P chart, as specified in Figure 7. Contrast this with the eight completely ineffective probes, which all fell outside this region of the diagram. Moreover, probes described by Betz et al. as binding “irreversibly,” or being “poor destaining,” or giving “high background,” or indeed as “toxic,” all fell in the “strongly PM bound” or “trapped in PM” regions of the AI-Log P chart. This constitutes further evidence that the QSAR model described in this paper is of wide applicability in defining the various PM probe types.

### 3.5. Possible Limitations and Extensions to the QSAR Model

Brief comments will now be offered on the relevance of the QSAR model when the lipid composition of the PM is unusual or specialised. As a case example, fluorescent probes for membrane rafts are considered, as these structures provide a well-studied instance of zones of the PM differing markedly in lipid composition. As previously mentioned, a single source document describing multiple raft probes was identified, namely a review article by Klymchenko and Kreder [32]. The structure parameters AI, HGH, and log P for all computable probes listed were generated; see Table S1. All 43 probes were small-molecule fluorochromes, the topic of the present analysis; however, some of these had only been used with model membrane systems, not with live cells. While a detailed analysis of these data is not possible in this brief tailpiece, some intriguing preliminary observations and comments can be offered.

Some probes discussed in [32] showed selective uptake into either liquid-ordered (Lo, such as the rafts) or liquid-disordered (Ld) membranes. Other probes showed no uptake selectivity but had the ability to report on properties that differ between Lo and Ld membranes, such as polarity or viscosity. All probes, however, were regarded as actual or potential PM-targeting compounds. Consequently, the first observation to note is that all probes (43/43), whether favouring Lo or Ld membranes, met the QSAR model criteria for PM binding. The marked difference in lipid composition between Lo and Ld membranes did not invalidate this relationship.

However, it seems that several other issues discussed by Klymchenko and Kreder can be addressed by the QSAR model. While this amounts to only a very partial analysis, it serves to illustrate the potential of the QSAR approach.

A first example considers which probes remain localised in the outer leaflet of the PM and exhibit slow flip-flop. The two dyes regarded in [32] as superior in this regard were di-4-ANEPPDHQ and NR12S. Both are predicted by the QSAR model to be trapped in the PM, the former dye because it is amphiphilic and possesses a very hydrophilic HG, and the latter because it is superamphiphilic. Consequently, both probes are predicted to exhibit slow flip-flops. The labelled lipid probe NBD-DOPE was also reported to exhibit a little flip-flop. Again, the QSAR model predicts trapping, and thus no fast flip-flop, due to the probe’s superamphiphilicity. Another way this topic can be addressed is to consider the dyes, which were stated to be quite unsuitable for selective labelling of the outer leaflet. Examples include R18 and derivatives of the cyanine dyes DiI and DiD. All these compounds are superlipophilic but not amphiphilic and are predicted by the QSAR model to partition into the hydrophobic membrane core. Consequently, they are not predicted to be retained in the outer leaflet.

Another topic addressed by Klymchenko and Kreder is membrane permeability and the loss of probes from the PM into the cell interior. In this context, probably non-specific cellular binding by PAH hydrocarbons was commented on. Therefore, it is of interest that of the five examples of unsubstituted PAHs listed, four have log P values in the range of 5–8. The QSAR model predicts such compounds will bind strongly to the PM but not be trapped in it, resulting in membrane permeability. The cellular internalisation of Laurdan and various of its derivatives were also mentioned. Laurdan itself was observed to be readily internalised, and, in keeping with this, the dye is very lipophilic but not amphiphilic. However, C-Laurdan, which possesses a terminal carboxylic acid substituent, was less permeable. The ionised form of this dye is superamphiphilic and is predicted to be trapped in the PM. Note, however, that the free acid species is predicted to be much more permeable. Klymchenko and Kreder also discussed a related pair of dyes with analogous properties, namely C-Laurdan-2 and S-Laurdan-2. The QSAR model predicts that while the former is strongly bound, it is only the latter that is trapped in the PM, in keeping with experimental observation.

Finally, we can consider whether the QSAR model could be tuned to predict probe selectivity for Lo or Ld membrane environments. The authors of [32] repeatedly emphasise that the relationship between dye behaviour in model membranes and in live cells is not always strong. Nevertheless, they repeatedly offer an Israelashvili-style stereochemical model to explain such selectivity. This suggests that probes that have cylindrical rather than conical lipophilic domains favour binding to Lo membranes. Consequently, we briefly comment on the possibility of simplistic modelling of this geometrical difference.

First, note that if the PAH dyes cited in the review are ranked on the basis of their length-to-width ratio (a crude measure of their cylindrical character), the reported preference for Lo membranes increases as the ratio increases. This holds even if the ratio is assessed merely by counting the numbers of C–C bonds in orthogonal directions. Another way of crudely assessing cylindrical vs. conical shape is possible in the case of lipids with fluorescent labels attached to the HG. The review’s authors describe marked steric effects, with a preference for Lo membranes falling as HG size increases. Thus, labels based on Rhodamine Lissamine or Texas Red result in Ld preference, with BODIPY or NBD labels resulting in a preference for Lo. If label size is modelled simplistically by summing the atomic weights of the component atoms, it is seen that Rhodamine Lissamine, Texas Red, and Atto dyes have “sizes” > 500 Daltons, whereas BODIPY and NBD are around 200.

Apart from the finding that the QSAR model does indeed predict the binding of probes to Lo and Ld regions of the PM, this section has largely been based on non-systematic snapshots. However, these suggest that a more extensive analysis might provide the basis for a useful, albeit simplistic, QSAR modelling approach and so allow an extension of the QSAR model presented in this current study.

## 4. Materials and Methods

The building bricks of the model—the integrated model, different aspects of which are described in Figure 2, Figure 3, Figure 5, Figure 6, Figure 7 and Figure 8—are based on material from [11] and papers cited in that source.

How structure parameters were estimated—the parameters required were AI, HGH, and log P; for details, see the above references. Briefly, in all cases, either the actual log P or the “nominal” log P values of the HG (i.e., HGH) and the lipophilic domain (i.e., AI) were estimated using the Hansch and Leo [4,22] fragment procedure. The cited sources provided examples of such estimations.

Data sets used for evaluating the predictions—while we needed to make such assessments on a wide range of chemical structures, we did not attempt to be encyclopedic in our coverage of PM probes described in the literature. Moreover, it was also necessary to avoid cherry-picking sets of compounds that might give a false impression of predictive success. To achieve these somewhat diverse aims, we identified source documents with the following features:

- Wide coverage of application areas was achieved by assembling sets of commercial surfactants, surface active drugs, saponins, fluorescent PM probes, and fluorescent PM probes of the styryl class.
- For each set of compounds, a single source document was sought that described at least a dozen compounds. Document types were varied and included a book chapter and a monograph, as well as review articles.
- Documents that contained either structural formulae of the compounds or that used nomenclature enabled easy retrieval of the structures from readily accessible sources.

Within each document, all compounds whose structure parameters could be estimated were then considered without exception. In all the sets of compounds, very large molecules and metal complexes were excluded. This was due to an inability of the Hansch and Leo [4,22] procedure to accurately estimate the structure parameters for such cases. Other probes whose estimates are likely to be imprecise are ampholytes and zwitterions and also those whose constituent resonance structures show markedly different ionic forms.

## 5. Conclusions

A simplistic decision-rule QSAR model has been devised that predicts the accumulation/non-accumulation of small-molecule fluorescent probes in the PM. The model was based on log P and various derived measures and enabled the roles of amphiphilicity, lipophilicity, and flip-flop to be assessed. The validity and generality of the model were demonstrated by evaluating its ability to predict amphiphilicity or PM accumulation patterns in surfactants, drugs, saponins, and probes. As the model is predictive, it will enable the design and synthesis of novel small-molecule PM probes to be carried out more efficiently. However, the model is simplistic and should be regarded as advisory, not prescriptive.

## Figures and Tables

**Table 1:** Various structural parameters of commercial surfactants. Examples from [14], chapter 1; see text for selection criteria. Tabulation follows the sequence of the chapter. AI: amphiphilicity index. HGH: headgroup hydrophilicity. Z: electric charge.

Surfactant Class or Example of Class	Structure Parameters		
	AI (Typical Chain Length of R)	HGH	Z
Ionised straight-chain fatty acids: R–CO_2_^−^ M^+^	5.8–11.2 (C10–20)	−5.2	1−
Linear alkyl-benzene sulfonates: R-C_6_H_4_-SO_3_^−^M^+^	8.5 (C12)	−4.5	1−
Long chain amines and their salts: R–NH_3_^+^ X^−^	5.5–8.7 (C12–18)	−2.2	0
		−3.4	1+
Quaternary ammonium salts, e.g., R–N+(CH_3_)_3_ X^−^	7.6 (C22, behenyl)	−4.1	1+
Quaternary ammonium salts, e.g., R–N+(CH_2_C_6_H_5_)(CH_3_)_2_ Cl^−^	5.2 (C16, cetyl)	−1.5	1+
*N*-alkyl pyrrolidones	4.7–8.5 (C8–12)	−1.0	0
Alkyl polyglycosides	5.8–7.9 (C10–14)	−3.4	0
β-*N*-Alkylaminopropionic acids: R^+^H_2_CH_2_CH_2_CO_2_^−^	5.5 (C12)	−3.6	0
α-Sulfofatty acid methyl esters: R–CH(SO_3_^−^)CO_2_CH_3_ M^+^	6.2–9.4 (C12–18)	−2.2	1−
*N*-Acyl-L-glutamates: R–CONH–CH(CO_2_^−^)CH_2_CH_2_CO_2_^−^ 2M^+^	6.2 and 10.1 (C12 and C18)	−7.2	2−
*N*-Lauryl sarcosinate: C_11_H_23_–CON(CH_3_)CH_2_CO_2_^−^ M^+^	6.3 (C11)	−3.2	1−

**Table 2:** Various structural parameters of surface-active drugs. Examples from [15], Table 1, and drug categories are those used by the authors; see text for selection criteria. AI: amphiphilicity index. HGH: headgroup hydrophilicity. Log P: the log of the octanol–water partition coefficient. Z: electric charge.

Name (Type of Drug)	Structure Parameters			
	AI	HGH	Log P	Z
Acetobutolol (antihypertensive)	1.9	−3.0	−1.1	1+
Adiphenine (anticholinergic)	4.0	−3.3	0.7	1+
Amitriptyline (antidepressant)	5.6	−4.0	1.6	1+
Bromodiphenylhydramine (antihistamine)	5.2	−3.9	1.3	1+
Chlorpromazine (antipsychotic)	4.2	−2.6	1.6	1+
Dextropropoxyphene (analgesic)	4.9	−3.5	1.4	1+
Dibucaine (local anesthetic)	3.9	−3.5	0.4	1+
Flupenthixol (tranquilizer)	5.2	−3.8	1.4	1+
Nortriptyline (antidepressant)	5.7	−3.7	2.0	1+
Penicillin G (antibiotic)	3.6	−4.2	−0.6	1−
Piperidolate (anticholinergic)	4.3	−5.2	−0.9	1
Sodium fusidate (antibiotic)	7.6	−6.3	1.3	1−
Tetracaine (local anesthetic)	3.0	−3.0	0.0	1+
Thiopental (general anesthetic)	4.5	−2.6	2.9	0
Trifluopromazine (antipsychotic)	4.7	−3.4	1.3	1+
Tripelennamine (antihistamine)	3.7	−2.8	0.9	1+

**Table 3:** Various structural parameters of saponins. Examples from the monograph of Abdelrahman and Jogaigh [16], names being those used in figure captions by the authors; see text for selection criteria. AI: amphiphilicity index. HGH: headgroup hydrophilicity. Z: electric charge.

Name	Structure Parameters		
	AI	HGH	Z
Aliospiroside A	8.6	−3.5	0
α-Chaconine	8.0	−3.5	0
Echynocystic acid	8.8	−3.6	1−
Gypsogenin	7.5	−3.2	1−
Phytolaccinic acid	7.8	−5.1	1−
Phytolaccinic acid, 23-O-Ac	8.7	−5.1	1−
Quillaic acid	6.1	−3.6	1−
Quillaic acid, 22β-OH	7.5	−5.2	1−
Soyasaponin Aa	8.3	−9.8	0
Soyasaponin βg	10.8	−6.5	0
Steroidal alkaloid saponin	9.3	−11.4	0
Steroidal saponin	5.6	−1.7	0
Triterpenoid saponin	9.7	−5.9	0

**Table 4:** Various structural parameters of plasma membrane probes. Examples from [1] and all structures that meet the selection criteria are included. AI: amphiphilicity index. HGH: headgroup hydrophilicity. Log P: the log of the octanol–water partition coefficient. NA: not applicable. Z: electric charge. Zw: zwitterion.

Probe Name	Structure Parameters				Cell Target of Probe
	AI	HGH	Log P	Z	
1	9.0	−1.1	7.9	0	Thiols
1P	14.0	−5.4	8.6	2−	Plasma membrane marker
8-TBT-8	8.3	−5.4	2.9	2+	Plasma membrane marker
ACal	6.3	−4.4	1.9	4−	Calcium ions
B-2AZ	16.0	−8.7	7.3	0 Zw	Plasma membrane marker
C-1	6.3	−3.7	3.5	1−	Membrane tension
C-2	7.0	−3.9	4.8	1−	Membrane tension
Calcium green C18	9.5	−10.2	−0.7	6−	Calcium ions
CL	8.1	−1.4	6.7	1−	Membrane tension
CS	9.5	−1.4	8.2	1−	Membrane tension
C18-Fura-2	10.1	−11.5	−1.4	5−	Calcium ions
C-Laurdan	8.1	−3.9	6.0	1−	Membrane microdomain
CL-Laurdan	8.1	−3.9	1.5	1−	Membrane microdomain
Di-4-ANEPPDHQ	8.0	−7.4	0.6	1+	Membrane microdomain
DSDMHDAB	9.0	−9.4	−0.4	2−	Nitric oxide
FD-9	NA	NA	14.8	0	Plasma membrane marker
Flu7	12.0	−8.6	3.4	2+	With Q12, enzyme activity
FC12SM	11.8	−5.0	6.8	0 Zw	Plasma membrane marker
F2N8	10.2	−4.6	5.6	1+	Membrane microdomain
F2N12S	11.0	−5.0	6.0	0 Zw	Plasma membrane marker
F2N12SM	11.8	−5.6	6.8	0 Zw	Plasma membrane marker
HGMem-3	NA	NA	16.6	0	Hg^2+^
HOCMem	NA	NA	18.0	0	HOCl
Laurdan	NA	NA	8.7	0	Plasma membrane marker
Mem-5	NA	NA	15.7	0	Cu^2+^
Mem-6	NA	NA	12.0	0	Cu^2+^
MemBright C3	14.7	−4.5	10.2	1+	Plasma membrane marker
MemBright C3.5	12.0	−4.5	12.6	1+	Plasma membrane marker
MemBright C5	12.0	−4.5	10.9	1+	Plasma membrane marker
MemBright C5.5	12.0	−4.5	13.3	1+	Plasma membrane marker
MemBright C7	12.0	−4.5	11.6	1+	Plasma membrane marker
MemBright C7.5	12.0	−4.5	14.0	1+	Plasma membrane marker
Mem-NO	12.3	−6.7	5.6	1+	Nitric oxide
Mem-SQAC	7.8	−5.1	2.7	1+	Plasma membrane marker
N4RA	8.4	−4.6	3.8	1−	Membrane microdomain
NR12S	12.2	−3.6	8.6	0 Zw	Membrane microdomain
PY3174	5.4	−7.2	−1.8	2+	Membrane microdomain
PY3184	5.5	−7.2	−1.7	2+	Membrane microdomain
PY3304	6.4	−6.8	−0.4	2+	Membrane microdomain
Q12	8.3	ca. −5	3.3	1−	With Flu-7, enzyme activity
SL2	5.4	−1.8	3.6	1−	Membrane microdomain
Structure 1	6.0	−6.1	−0.1	4−	Thioredoxin
TPE-MEM	14.7	−9.6	5.1	2+	Plasma membrane marker
TPE-Py-EEGTIGYG	10.0	−10.1	−0.1	2−	Cu^2+^
TTVP	6.7	−6.8	−0.1	2+	Plasma membrane marker
ZTRS-C12	NA	NA	8.6	0	Zn^2+^

## Data Availability

All data generated or analysed during this study are included in the published article.

## Supplementary Materials

The following supporting information can be downloaded at: https://www.mdpi.com/article/10.3390/molecules28227589/s1; Figure S1 Exemplar structures of amphiphilic compounds listed in Table 1, Table 2, Table 3 and Table 4, illustrating the chemical diversity of such molecules; Table S1 Various structure parameters of fluorescent probes for membrane rafts, plus information concerning predicted cell localisation and predicted intracellular mobility of probes.
