# Supplementary material for: How to Target Small-Molecule Fluorescent Imaging Probes to the Plasma Membrane—The Influence and QSAR Modelling of Amphiphilicity, Lipophilicity, and Flip-Flop
Source: Molecules. 2023 Nov 14;28(22):7589. doi: 10.3390/molecules28227589 (PMC10674381; doi:10.3390/molecules28227589)
Supplement: Supplementary file 1 [file molecules-28-07589-s001.zip › molecules-2651795-supplementary.pdf]

How to target small-molecule fluorescent imaging probes to the plasma membrane: The influence and QSAR modelling of amphiphilicity, lipophilicity and flip-flop.

Richard W Horobin and Juan C Stockert

Supplementary material

Figure S1. Exemplar structures of amphiphilic compounds listed in Tables 1–4, illustrating the chemical diversity of such molecules. (A) Capric acid sodium salt (commercial surfactant). (B) *N*-Octyl pyrrolidone (commercial surfactant). (C) C-Laurdan (fluorescent probe). (D) Chlorpromazine (drug). (E) Triterpenoid saponin. (F) Steroidal saponin. (G) Penicillin G (drug). (H) Calcium green C18 (fluorescent probe).

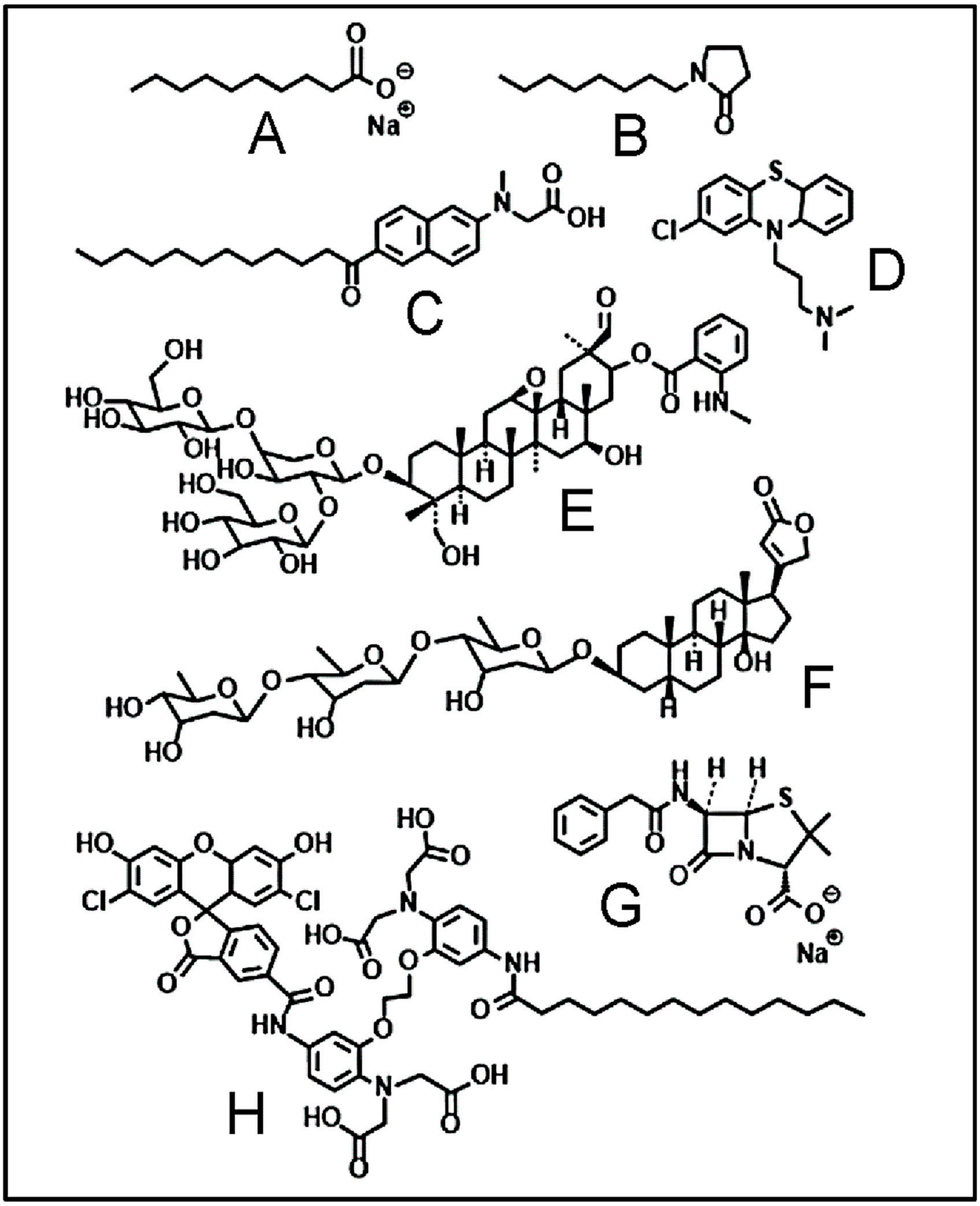

Table S1. Various structure parameters of fluorescent probes for membrane rafts, plus information concerning predicted cell localisation and predicted intracellular mobility of probes. Examples from [36]. Naming and sequencing of probes as in the data source. Included are all structures which meet the inclusion criteria. AI: amphiphilicity index. HGH: headgroup hydrophilicity. HGS: headgroup size. NA: not applicable. Log P: the log of the octanol-water partition coefficient. PM: plasma membrane.

Name of probe	AI	HGH	Log P	Predicted localisation & mobility
Cholestatrienol	8.9	-1.6	7.3	PM<br>Trapped
NBD-chol	NA	NA	6.4	PM<br>Not trapped
TF-chol	NA	NA	10.2	PM<br>Trapped
5-BODIPY-PC	8.5	-1.7	6.8	PM<br>Trapped
12-NBD-PC	9.6	-1.7	7.9	PM<br>Trapped
5-BODIPY-SM	9.7	-2.6	7.1	PM<br>Trapped
12-BODIPY-SM	13.5	-2.6	10.9	PM<br>Trapped
6-NBD-SM	8.9	-2.6	6.3	PM<br>Trapped
12-NBD-SM	10.9	-2.6	8.3	PM<br>Trapped
X-DPPE NBD	9.5	-1.0	8.5	PM<br>Trapped
X-DPPE Lissamine Rhodamine	NA	NA	22.2	PM<br>Trapped
X-DPPE Texas Red	NA	NA	21.8	PM<br>Trapped
X-DOPE NBD	17.4	-1.0	16.4	PM<br>Trapped
X-DOPE Lissamine Rhodamine	NA	NA	20.6	PM<br>Trapped
X-DOPE Texas Red	NA	NA	20.2	PM<br>Trapped
4-Atto647N-SM	13.4	-2.1	11.3	PM<br>Trapped
4-Atto532-SM	7.4	-4.0	3.4	PM<br>Trapped

SM-Atto532	13.7	-3.1	10.6	PM<br>Trapped
DiI-C12	NA	NA	12.3	PM<br>Trapped
DiI-C16	NA	NA	15.7	PM<br>Trapped
DiI-C21	NA	NA	19.1	PM<br>Trapped
DiI-C22	NA	NA	20.8	PM<br>Trapped
Fast DiO	NA	NA	12.5	PM<br>Trapped
DiD-C18	NA	NA	17.9	PM<br>Trapped
R18	NA	NA	12.6	PM<br>Trapped
DPH	NA	NA	6.0	PM<br>Not trapped
LcTMA-DPH	13.1	-4.1	9.0	PM<br>Trapped
NAP	NA	NA	7.3	PM<br>Not trapped
Perylene	NA	NA	6.2	PM<br>Not trapped
Terrylene	NA	NA	8.5	PM<br>Trapped
Rubicene	NA	NA	7.8	PM<br>Not trapped
Laurdan	NA	NA	8.4	PM<br>Trapped
C-Laurdan	11.8	-3.9<br>Carboxylate	7.9	PM<br>Trapped
di-4-ANEPPDHQ	5.4	-6.7	-1.3	PM<br>Trapped
F2N12S	11.0	-3.1	7.9	PM<br>Trapped
F66NS	16.1	-2.3	13.8	PM<br>Trapped
NR12S	12.9	-3.6	9.3	PM<br>Trapped
FCVJ	NA	NA	7.4	PM<br>Not trapped
5-CCVJ-PC	10.1	-1.8	8.3	PM<br>Trapped
DMPC-DDCJ	14.7	-2.7	12.0	PM<br>Trapped

C-Laurdan-2	9.2	-4.0	5.2	PM<br>Trapped
S-Laurdan-2	10.0	-4.5	5.5	PM<br>Trapped
BODIPY-Ph-C12	10.2	-3.1	7.1	PM<br>Trapped
